# *Cannabis sativa*: The Plant of the Thousand and One Molecules

**DOI:** 10.3389/fpls.2016.00019

**Published:** 2016-02-04

**Authors:** Christelle M. Andre, Jean-Francois Hausman, Gea Guerriero

**Affiliations:** Environmental Research and Innovation, Luxembourg Institute of Science and TechnologyEsch-sur-Alzette, Luxembourg

**Keywords:** fibers, hemp, *Cannabis*, cellulose, lignin, cannabinoids, terpenes, lignans

## Abstract

*Cannabis sativa* L. is an important herbaceous species originating from Central Asia, which has been used in folk medicine and as a source of textile fiber since the dawn of times. This fast-growing plant has recently seen a resurgence of interest because of its multi-purpose applications: it is indeed a treasure trove of phytochemicals and a rich source of both cellulosic and woody fibers. Equally highly interested in this plant are the pharmaceutical and construction sectors, since its metabolites show potent bioactivities on human health and its outer and inner stem tissues can be used to make bioplastics and concrete-like material, respectively. In this review, the rich spectrum of hemp phytochemicals is discussed by putting a special emphasis on molecules of industrial interest, including cannabinoids, terpenes and phenolic compounds, and their biosynthetic routes. Cannabinoids represent the most studied group of compounds, mainly due to their wide range of pharmaceutical effects in humans, including psychotropic activities. The therapeutic and commercial interests of some terpenes and phenolic compounds, and in particular stilbenoids and lignans, are also highlighted in view of the most recent literature data. Biotechnological avenues to enhance the production and bioactivity of hemp secondary metabolites are proposed by discussing the power of plant genetic engineering and tissue culture. In particular two systems are reviewed, i.e., cell suspension and hairy root cultures. Additionally, an entire section is devoted to hemp trichomes, in the light of their importance as phytochemical factories. Ultimately, prospects on the benefits linked to the use of the *-omics* technologies, such as metabolomics and transcriptomics to speed up the identification and the large-scale production of lead agents from bioengineered *Cannabis* cell culture, are presented.

## Introduction

The current climatic and economic scenario pushes toward the use of sustainable resources to reduce our dependence on petrochemicals and to minimize the impact on the environment. Plants are precious natural resources, because they can supply both phytochemicals and lignocellulosic biomass. In this review, we focus on hemp (*Cannabis sativa* L.), since it is a source of fibers, oil and molecules and as such it is an emblematic example of a multi-purpose crop. We treat the aspects related to the use of hemp biomass and, more extensively, those linked to its wide variety of phytochemicals.

Known since the ancient times for its medicinal and textile uses (Russo et al., 2008; Skoglund et al., 2013), hemp is currently witnessing a revival, because of its rich repertoire of phytochemicals, its fibers and its agricultural features, namely quite good resistance to drought and pests, well-developed root system preventing soil erosion, lower water requirement with respect to other crops, e.g., cotton. This shows the great versatility of this fiber crop and encourages future studies focused on both *Cannabis* (bio)chemistry and genetic engineering. Hemp varieties producing oil, biomass or even both are currently cultivated and the availability of the hemp genome sequence greatly helps molecular studies on this important crop (van Bakel et al., 2011). In addition, the scientific community is very much interested in harnessing *Cannabis* pharmacological power: for example microorganisms are being engineered to produce Δ^9^-tetrahydrocannabinolic acid (THCA) and cannabidiolic acid (CBDA) (Taura et al., 2007a; Zirpel et al., 2015).

The final scope of this review is to discuss the potential of hemp for industry and to highlight its importance for the bio-economy. More specifically, we: (i) describe the use of hemp biomass (i.e., the fibers), (ii) discuss hemp molecules of industrial interest (namely cannabinoids, terpenes and phenolic compounds), (iii) describe the potential of hemp trichomes as pharma-factories and (iv) discuss the potential of genetic engineering, by describing the use of plant cell suspension and hairy root cultures.

## Hemp Stem: A Source of Fibers with Antibacterial Properties

**FIGURE 1:**
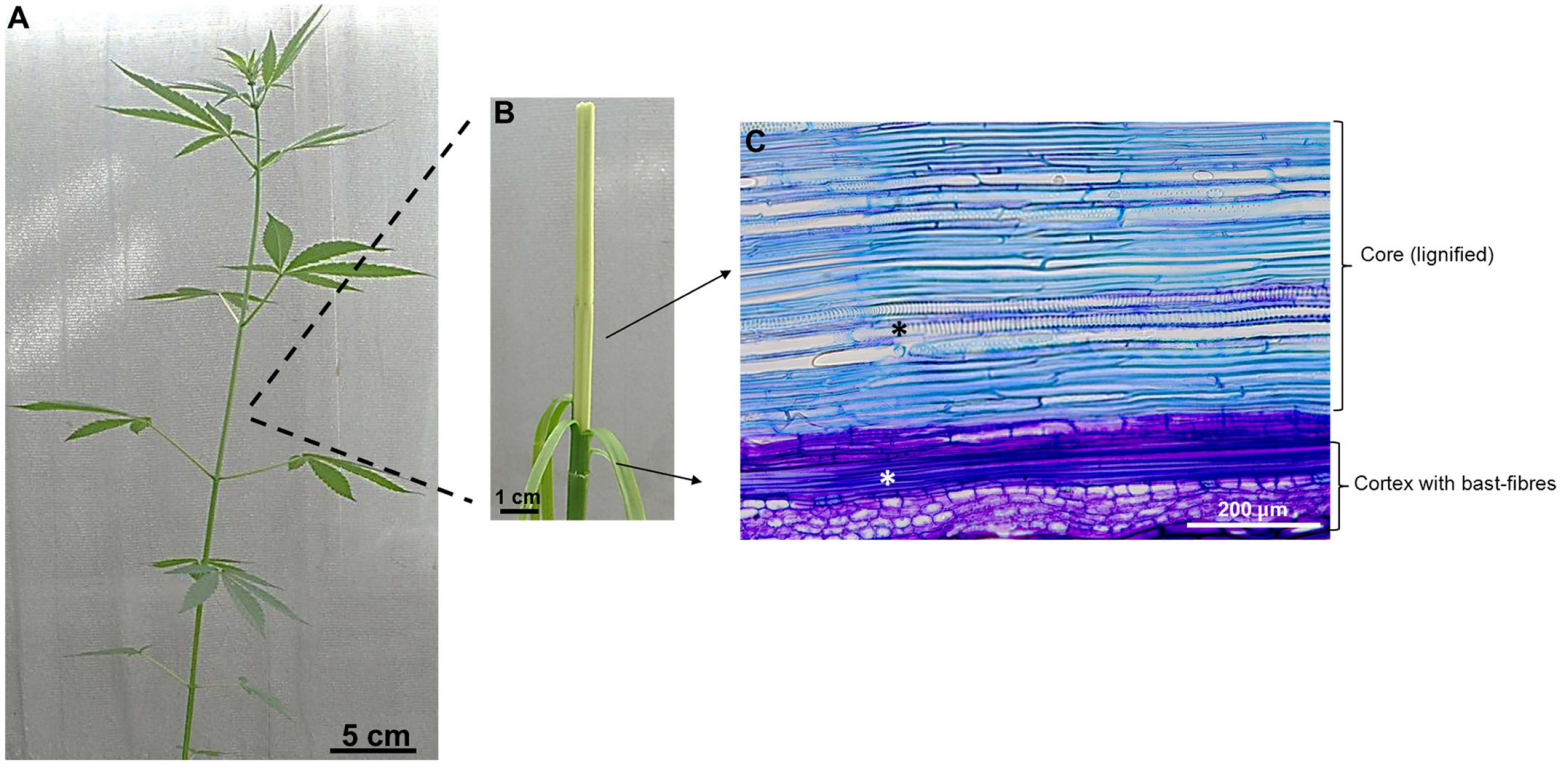
**Anatomical details of *Cannabis* stem. (A)** Stem of an adult plant (ca 2 months); **(B)** The stem can be peeled off and shows a lignified core and a cortex with bast fibers. **(C)** Longitudinal section of hemp stem stained with toluidine blue showing the cortex with a bundle of bast-fibers (white asterisk) and the core with xylem vessels (black asterisk).

Plant lignocellulosic biomass is an abundant renewable resource, which can provide biopolymers, fibers, chemicals and energy (Guerriero et al., 2014, 2015, 2016). Trees are important for the provision of wood, however, also fast-growing herbaceous species, like textile hemp (which has a THC content <0.3%; Weiblen et al., 2015), can provide high biomass quantities in a short time. The stem of this fiber crop supplies both cellulosic and woody fibers: the core is indeed lignified, while the cortex harbors long cellulose-rich fibers, known as bast fibers (**Figure 1**) (Guerriero et al., 2013).

This heterogeneous cell wall composition makes hemp stem an interesting model to study secondary cell wall biosynthesis, in particular the molecular events underlying the deposition of cortical gelatinous bast fibers and core woody fibers.

*Cannabis* woody fibers (a.k.a “hurds” or “shivs”) are used for animal bedding because of their high absorption capacity and for the creation of a concrete-like material.

Hemp bast fibers are used in the biocomposite sector as a substitute of glass fibers. The automotive industry is particularly keen on using hemp bast fibers to produce bioplastics: this material is stronger than polypropylene plastic and lighter in weight (Marsh, 2003).

Beyond the applications in the construction and automotive industries, hemp fibers are attractive also in the light of their natural antibacterial property. Hemp bast fibers have been indeed described as antibacterial (Hao et al., 2014; Khan et al., 2015) and their use for the manufacture of an antibacterial finishing agent (Bao et al., 2014), surgical devices (Gu, 2006) or functionalized textiles (Cassano et al., 2013) has been reported. This property is linked to the chemical composition of hemp bast fibers: both free and esterified sterols and triterpenes have been identified, among which β-sitosterol and β-amyrin (Gutiérrez and del Río, 2005). These compounds possess known antibacterial properties (Kiprono et al., 2000; Ibrahim, 2012). Hemp bast fibers were also found to contain cannabinoids (2% of the total metabolite extract) (Bouloc et al., 2013 and references therein). More recently hemp hurd powder showed antibacterial properties against *Escherichia coli* (Khan et al., 2015). Since the hurd has a higher lignin content than the bast fibers, its antibacterial property may be linked to lignin-related compounds such phenolic compounds, as well as alkaloids and cannabinoids (Appendino et al., 2008; Khan et al., 2015).

## Hemp Phytochemicals: Their Production Pathways and Myriad of Biological Activities

Numerous chemicals are produced in hemp through the secondary metabolism. They include cannabinoids, terpenes and phenolic compounds (Flores-Sanchez and Verpoorte, 2008) and will be further described in the next sections. Although the pharmacological properties of cannabinoids have extensively been studied and are the most recognized hemp bioactives, the other components have no reasons to envy them, as they have also been associated with potent health-promoting properties. Research on *Cannabis* phytochemicals, as well as the widespread therapeutic use of *Cannabis* products, has been limited due to various reasons, including illegality of cultivation (due to its psychoactivity and potential for inducing dependence), variability of active components, and low abundance of some of them *in planta*. Further attentions is now drawn toward non-THC *Cannabis* active components, which may act synergistically and contribute to the pharmacological power and entourage effects of medicinal-based *Cannabis* extract (Russo, 2011).

### Phytocannabinoids

Phytocannabinoids represent a group of C21 or C22 (for the carboxylated forms) terpenophenolic compounds predominantly produced in *Cannabis.* They have also been reported in plants from the *Radula* and *Helichrysum* genus (Appendino et al., 2008) but our knowledge on non-*Cannabis* source of cannabinoids is still in its infancy (Gertsch et al., 2010). More than 90 different cannabinoids have been reported in the literature, although some of these are breakdown products (ElSohly and Slade, 2005; Brenneisen, 2007; Radwan et al., 2009; Fischedick et al., 2010) and they are generally classified into 10 subclasses (Brenneisen, 2007). In this review, we will focus on the most abundant compounds found in the drug- and fiber-type *Cannabis*. The predominant compounds are THCA, CBDA and cannabinolic acid (CBNA), followed by cannabigerolic acid (CBGA), cannabichromenic acid (CBCA) and cannabinodiolic acid (CBNDA) (ElSohly and Slade, 2005). THCA is the major cannabinoid in the drug-type *Cannabis*, while CBDA predominates in fiber-type hemps. CBCA has been reported to dominate in the cannabinoid fraction of young plants and to decline with maturation (Meijer et al., 2009). The phytocannabinoid acids are non-enzymatically decarboxylated into their corresponding neutral forms, which occur both within the plant and, to a much larger extent, upon heating after harvesting (Flores-Sanchez and Verpoorte, 2008). Phytocannabinoids accumulate in the secretory cavity of the glandular trichomes, which largely occur in female flowers and in most aerial parts of the plants, as further described in the next section. They have also been detected in low quantity in other parts of the plants including the seeds (Ross et al., 2000), roots (Stout et al., 2012) and the pollen (Ross et al., 2005), in an extent depending on the drug- or fiber-type of *Cannabis*, as described in **Table 1**. More generally, the concentration of these compounds depends on tissue type (**Table 1**), age, variety, growth conditions (nutrition, humidity, light level), harvest time and storage conditions (Khan et al., 2014). The level of phytocannabinoids in hempseeds, and thereby of hempseed oil, should be very low as the kernel contains only trace amount of THC or CBD (Leizer et al., 2000; Ross et al., 2000). However, higher THC concentrations are found on the outside surface of the seed coat, possibly as the result of contamination with plant leaves or flowers (Ross et al., 2000). Recently, significant amounts of cannabinoids, and particularly of THC, were found in five out of 11 hempseed oil samples available on the Croatian market, suggesting that both contaminations are due to improper processing procedures and the illegal use of drug-type hemp (with a THC + CBN/CBD ratio >1) for nutritional purposes (Petrović et al., 2015). Cannabinoids in the leaves have been shown to decrease with the age and along the stem axis, with the highest levels observed in the leaves of the uppermost nodes (Pacifico et al., 2008). Cannabinoid contents in the stem are scarce in the literature. An analysis performed on the dust obtained from the top section of the stem of fiber-type hemp (low percentage of bast fibers) revealed a low THC and CBD content (0.04 and 1.3% on average, respectively) (Cappelletto et al., 2001). Kortekaas et al. (1995) analyzed the cannabinoid content of hemp black liquor. The sum of the THC and CBD fractions (without reporting the distinct amounts of each of them) in hemp stem wood and bark extractives was 2 and 1%, respectively, which represented 0.003 and 0.0005% of the total fiber content.

**Table 1:** Summary of the concentrations in cannabinoids found in different parts of the hemp plants, *in vitro* hairy roots, and some commercial medicinal products.

	Hairy roots	Root		Seed		Stem		Leaves		Pollen		Flower		^∗^Bedrocan^®^	^∗^Bediol^®^
Molecules		Fiber-type	Drug-type	Fiber-type	Drug-type	Fiber-type	Drug-type	Fiber-type	Drug-type	Fiber-type	Drug-type	Fiber-type	Drug-type	Drug-type	Drug-type
THC	1.04**^a^**			0–12 (<0.5 in kernel)^c^ 3–29^d^	36–174 (<2 in kernel)^c^ 15–70^d^	196–475^j^	3000^e^	2000^f^	60300^g^ 22000^f^ 8000^e^		31230^h^	76300^i^	95100^g^ 34000–200000^i^ 152000^e^	190000^i^	19000^i^
CBD	1.67**^a^**	14.3^b^		67–244^d^	4.2–78^d^	179^b^ 7850–18090^j^		1790^b^ 20000^f^	11200^g^ 3000^f^		440^h^	8590^b^ 6000^i^	10900^g^ <600^i^	<600^i^	79800^i^
CBN				2–7^d^	3.4–8.4^d^	0–47^j^			800^g^		1350^h^		600^g^		
CBG	1.63**^a^**							2000^f^	1000^f^		1310^h^	<600^i^	1000–10000^i^	11200^i^	1700^i^
THCV											510^h^	<600^i^	(<600) – 1300^i^	1300^i^	<600^i^
CBC											3240^h^	4 600^i^	900–2200^i^	2300^i^	5400^i^

*Data are expressed in μg g^*-1*^ of dry weight. The most recent references have been used, when available. Abbreviations: THC, Δ^*9*^-tetrahydrocannabinol; CBD, cannabidiol; CBN, cannabinol; CBG, cannabigerol; THCV, tetrahydrocannabivarin; CBC, cannabichromene. References: ^a^Farag and Kayser, 2015; ^b^Adapted from Stout et al., 2012; ^c^Ross et al., 2000; ^d^Petrović et al., 2015 (concentration in hempseed oil); ^e^Potter, 2004; ^f^Pacifico et al., 2008 (growth curve experiment, the maximum concentrations are represented); ^g^Bruci et al., 2012; ^h^Ross et al., 2005; ^i^Fischedick et al., 2010; ^j^Cappelletto et al., 2001, data from stem dust. ^∗^Commercial pharmaceutical preparations.*

### Biosynthetic Pathway Leading to Phytocannabinoids

**FIGURE 2:**
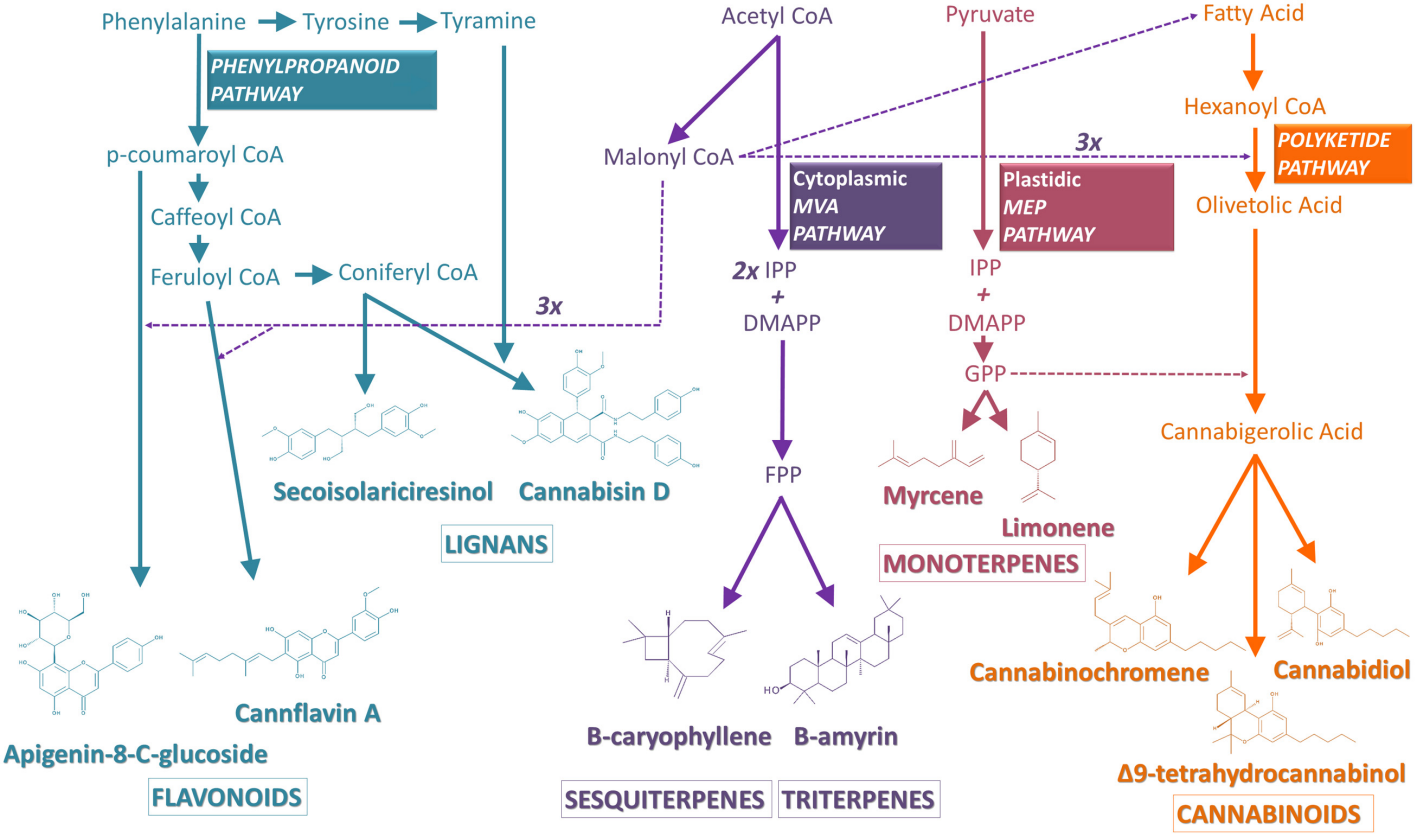
**Schematic view of the biosynthetic pathways leading to the *Cannabis* secondary metabolites discussed in this review.** Transport of precursors is represented by dashed arrows, while direct catalytic reactions are depicted by bold arrows. See text for detailed pathways. Abbreviations used: IPP, isopentenyl diphosphate; DMAPP, dimethylallyl diphosphate; GPP, geranyl diphosphate; FPP, farnesyl diphosphate; MVA, mevalonate; MEP, methylerythritol phosphate.

The biosynthesis of cannabinoids from *C. sativa* has only been recently elucidated. The precursors of cannabinoids actually originate from two distinct biosynthetic pathways: the polyketide pathway, giving rise to olivetolic acid (OLA) and the plastidal 2-C-methyl-D-erythritol 4-phosphate (MEP) pathway, leading to the synthesis of geranyl diphosphate (GPP) (Sirikantaramas et al., 2007) (**Figure 2**). OLA is formed from hexanoyl-CoA, derived from the short-chain fatty acid hexanoate (Stout et al., 2012), by aldol condensation with three molecule of malonyl-CoA. This reaction is catalyzed by a recently discovered polyketide synthase (PKS) enzyme and an olivetolic acid cyclase (OAC) (Gagne et al., 2012). The geranylpyrophosphate:olivetolate geranyltransferase catalyzes the alkylation of OLA with GPP leading to the formation of CBGA, the central precursor of various cannabinoids (Fellermeier and Zenk, 1998). Three oxidocyclases will then be responsible for the diversity of cannabinoids: the THCA synthase (THCAS) converts CBGA to THCA, while CBDA synthase (CBDAS) forms CBDA and CBCA synthase (CBCAS) produces CBCA (Sirikantaramas et al., 2004, 2005; Taura et al., 2007b). Propyl cannabinoids (cannabinoids with a C3 side-chain, instead of a C5 side-chain), such as tetrahydrocannabivarinic acid (THCVA), synthetized from a divarinolic acid precursor, have also been reported in *Cannabis* (Flores-Sanchez and Verpoorte, 2008).

### Health Benefits Linked to Cannabinoids

The pharmacology of phytocannabinoids has previously been reviewed elsewhere (Pacher et al., 2006; Russo, 2011; Hill et al., 2012; Giacoppo et al., 2014; Burstein, 2015) and a brief summary and update will be presented hereafter.

Most of the biological properties related to cannabinoids rely on their interactions with the endocannabinoid system in humans. The endocannabinoid system includes two G protein-coupled cannabinoid receptors, CB1 and CB2, as well as two endogenous ligands, anandamide and 2-arachidonylglycerol. Endocannabinoids are thought to modulate or play a regulatory role in a variety of physiological processing including appetite, pain-sensation, mood, memory, inflammation, insulin, sensitivity and fat and energy metabolism (De Petrocellis et al., 2011; Di Marzo and Piscitelli, 2015). The psychoactive decarboxylated form of THCA, THC, is a partial agonist of both CB1 and CB2 receptors, but has higher affinity for the CB1 receptor, which appears to mediate its psychoactive properties. In addition to being present in the central nervous system and throughout the brain, CB1 receptors are also found in the immune cells and the gastrointestinal, reproductive, adrenal, heart, lung and bladder tissues, where cannabinoids can therefore also exert their activities. CB2 receptors are thought to have immunomodulatory effects and to regulate cytokine activity. But THC has actually more molecular targets than just CB1 and CB2 receptors, and exhibit potent anti-inflammatory, anti-cancer, analgesic, muscle relaxant, neuro-antioxidative (De Petrocellis et al., 2011), and anti-spasmodic activities (Pacher et al., 2006). However, THC has been also associated with a number of side effects, including anxiety, cholinergic deficits, and immunosuppression (Russo, 2011). CBDA is the most prevalent phytocannabinoid in the fiber-type hemp, and the second most important in the drug chemotypes. CBD (decarboxylation of CBDA) presents a large array of pharmacological properties, as recently reviewed in Burstein (2015), which has been downplayed for many years, as compared to THC. CBD acts yet as an important entourage compound as it is able to reduce the side effects of THC (Englund et al., 2012), and may thereby increase the safety of *Cannabis*-based extracts. CBD itself has been shown in *in vitro* and animal studies to possess, among others, anti-anxiety, anti-nausea, anti-arthritic, anti-psychotic, anti-inflammatory, and immunomodulatory properties (Burstein, 2015). CBD is a very promising cannabinoid as it has also shown potential as therapeutic agents in preclinical models of central nervous system diseases such as epilepsy, neurodegenerative diseases, schizophrenia, multiple sclerosis, affective disorders and the central modulation of feeding behavior (Hill et al., 2012). Interestingly, CBD presents also strong anti-fungal and anti-bacterial properties, and more interestingly powerful activity against methicillin-resistant *Staphylococcus aureus* (MRSA) (Appendino et al., 2008). After THC and CBD, CBC is the third most prevalent phytocannabinoid. CBC presents notably anti-inflammatory (Delong et al., 2010), sedative, analgesic (Davis and Hatoum, 1983), anti-bacterial and antifungal properties (Eisohly et al., 1982). CBC is also a potent inhibitor of anandamide uptake, an endogenous ligand of CB receptors (De Petrocellis et al., 2011). CBN is a degradation product of THC and is mostly found in aged *Cannabis*. CBN has a twofold lower affinity for CB1 receptors and a threefold higher affinity for the CB2 receptors, as compared to THC. It thus affects cells of the immune system more than the central nervous system, as reviewed in (McPartland and Russo, 2001). Current cannabinoid-based therapeutic treatments is limited to special cases, i.e., spasticity associated to multiple sclerosis in adult patients, to treat nausea/vomiting linked to cancer therapies, to stimulate appetite in HIV-positive patients (Giacoppo et al., 2014; Lynch and Ware, 2015). Borrelli et al. (2013), after highlighting the beneficial effects of CBG on murine colitis, suggest that this cannabinoid should also be considered for clinical experimentation in patients affected by inflammatory bowel disease.

### Adverse Health Effects of Cannabinoids

As mentioned earlier, the recreational and medical use of *Cannabis* as well as of THC and other synthetic cannabinoids have also been associated with numerous side effects. Two recent reviews (Volkow et al., 2014; van Amsterdam et al., 2015) notably reported the adverse health effects linked to the use of natural *Cannabis* and synthetic cannabinoids, respectively. When adjusted for confounders such as cigarette smoking, the impact of short- and long-term use appear to be similar for both types of consumption and are directly linked to the level of THC or its synthetic analog. The THC content of recreational *Cannabis* has indeed drastically increased in the last 30 years (from 3% in 1980s to almost 20% now, as reported in **Table 1**), with very low level of the other cannabinoids such as CBD. Effects of short-term use include memory and cognitive deficits, impaired motor coordination, and psychosis. Long-term use of THC has been associated to an increased risk of addiction, cognitive impairment, altered brain development when initial use was done early in adolescence, and an increased risk of chronic psychosis disorder including schizophrenia. The protective role that CBD could play to alleviate these negative effects is now well established and documented (Iseger and Bossong, 2015).

### Terpenes

Terpenes form the largest group of phytochemicals, with more than 100 molecules identified in *Cannabis* (Rothschild et al., 2005; Brenneisen, 2007). Terpenes are responsible for the odor and flavor of the different *Cannabis* strains. They have therefore likely contributed to the selection of *Cannabis* narcotic strains under human domestication (Small, 2015). Terpenes are classified in diverse families according to the number of repeating units of 5-carbon building blocks (isoprene units), such as monoterpenes with 10 carbons, sesquiterpenes with 15 carbons, and triterpenes derived from a 30-carbon skeleton. Terpene yield and distribution in the plant vary according to numerous parameters, such as processes for obtaining essential oil, environmental conditions, or maturity of the plant (Meier and Mediavilla, 1998; Brenneisen, 2007). Mono- and sesquiterpenes have been detected in flowers, roots, and leaves of *Cannabis*, with the secretory glandular hairs as main production site. Monoterpenes dominate generally the volatile terpene profile (from 3.1 to 28.3 mg g^-1^ of flower dry weight, Fischedick et al., 2010) and include mainly D-limonene, β-myrcene, α- and β-pinene, terpinolene and linalool. Sesquiterpenes, and β-caryophyllene and α- humulene in particular, occur also to a large extent in *Cannabis* extracts (from 0.5 to 10.1 mg g^-1^ of flower dry weight, Fischedick et al., 2010). Triterpenes have also been detected in hemp roots, as friedelin and epifriedelanol (Slatkin et al., 1971), in hemp fibers as β-amyrin (Gutiérrez and del Río, 2005) and in hempseed oil as cycloartenol, β-amyrin, and dammaradienol (Paz et al., 2014).

Terpenes, along with cannabinoids, have successfully been used as chemotaxonomic markers in *Cannabis*, as they are both considered as the main physiologically active secondary metabolites (Fischedick et al., 2010; Elzinga et al., 2015). When grown in standardized conditions, a significant and positive correlation was found between the level of terpenes and cannabinoids (Fischedick et al., 2010). This may be explained by the fact that mono- and sesquiterpenes are synthesized in the same glandular trichomes in which the cannabinoids are produced (Meier and Mediavilla, 1998). This association was, however, not confirmed on a larger panel of samples coming from different origins (Elzinga et al., 2015).

### Biosynthetic Pathways Leading to the Different Classes of Terpenes

Two different biosynthetic pathways contribute, in their early steps, to the synthesis of plant-derived terpenes (**Figure 2**). Whereas the cytosolic mevalonic acid (MVA) pathway is involved in the biosynthesis of sesqui-, and tri-terpenes, the plastid-localized MEP pathway contributes to the synthesis of mono-, di-, and tetraterpenes (Bouvier et al., 2005). MVA and MEP are produced through various and distinct steps, from two molecules of acetyl-coenzyme A and from pyruvate and D-glyceraldehyde-3-phosphate, respectively. They are further converted to isopentenyl diphosphate (IPP) and isomerised to dimethylallyl diphosphate (DMAPP), the end point of the MVA and MEP pathways. In the cytosol, two molecules of IPP (C5) and one molecule of DMAPP (C5) are condensed to produce farnesyl diphosphate (FPP, C15) by farnesyl diphosphate synthase (FPS). FPP serves as a precursor for sesquiterpenes (C15), which are formed by terpene synthases and can be decorated by other various enzymes. Two FPP molecules are condensed by squalene synthase (SQS) at the endoplasmic reticulum to produce squalene (C30), the precursor for triterpenes and sterols, which are generated by oxidosqualene cyclases (OSC) and are modified by various tailoring enzymes. In the plastid, one molecule of IPP and one molecule of DMAPP are condensed to form GPP (C10) by GPP synthase (GPS). GPP is the immediate precursor for monoterpenes (Kempinski et al., 2015).

### Health Benefits Associated with Terpenes

Terpenes are lipophilic compounds that easily cross membranes and the blood-brain barrier in particular (Fukumoto et al., 2006). They present a wide-array of pharmacological properties, which have recently been described in several reviews (Russo, 2011; Singh and Sharma, 2015). The biological activities of D-limonene, also commonly found in *Citrus* essential oils, have been well described in the literature. It notably exhibits potent anti-cancer, anxiolytic and immunostimulating properties in humans (Komori et al., 1995). β-myrcene, a terpene commonly found in hop, is recognized as a potent anti-inflammatory, analgesic, and anxiolytic component (Cleemput et al., 2009). α-Pinene is an acetylcholinesteral inhibitor, and may thereby aid memory abilities (Kennedy et al., 2011), which could counteract the memory deficits induced by THC. Linalool, commonly found in *Lavandula angustifolia*, possesses similar properties to the ones described for its monoterpene counterparts, i.e., analgesic, anti-anxiety, anti-inflammatory, and anticonvulsant (Russo, 2011). β-caryophyllene, a well-known active principle of black pepper and Copaiba balsam, possesses potent anti-inflammatory and gastric cytoprotector activities (Singh and Sharma, 2015). Interestingly, it selectively binds to the CB2 receptor and could therefore technically be considered as a phytocannabinoid (Gertsch et al., 2008). Pentacyclic triterpenes such as β-amyrin and cycloartenol have been shown to possess numerous biological activities including anti-bacterial, anti-fungal, anti-inflammatory and anti-cancer properties (Vázquez et al., 2012; Moses et al., 2013). These triterpenes are key contributors to the pharmacological properties of numerous medicinal herbs (Kirby et al., 2008; Yadav et al., 2010; Sawai and Saito, 2011).

### Phenolic Compounds

Phenolic compounds, also known as phenylpropanoids, constitute one of the most widely distributed group of secondary metabolites in the plant kingdom. They present more than 10,000 different structures, including phenolic acids, such benzoic and hydroxycinnamic acids, flavonoids such as flavonols and flavones, stilbenes and lignans (Andre et al., 2010). In *Cannabis*, about 20 flavonoids have been identified, mainly belonging to the flavone and flavonol subclasses (Flores-Sanchez and Verpoorte, 2008). These include the *O*-glycoside versions of the aglycones apigenin, luteolin, kaempferol and quercetin, as well as cannflavin A and cannflavin B, which are methylated isoprenoid flavones that are unique to *Cannabis* (**Figure 2**) (Ross et al., 2005). Phenolic amides and lignanamides have also been described in *Cannabis* fruits and roots (Sakakibara et al., 1992; Lesma et al., 2014). The lignanamides belong to the lignan class of compounds and include cannabisin-like compounds (of the types A-, B-, C-, D-, E-, F-, and G) and grossamide (Flores-Sanchez and Verpoorte, 2008). Similar compounds such as cannabisin D, have been described in *Cannabis* leaves, where it was strongly induced upon the UV-C treatment (Marti et al., 2014). Interesting amounts of lignans were recently found in the hydrophilic extract of hemp seeds. The hemp seed lignan profile was shown to be dominated by syringaresinol and medioresinol, followed by secoisolariciresinol, lariciresinol, and pinoresinol (Smeds et al., 2012). Hemp seeds contain, however, about 20-times less total lignans (32 mg of total lignans per 100 g of dry weight) than flax seeds, a well-known source of lignans. Interestingly, the lignan content of hulled hemp seeds represents only 1% of the content in whole seed (Smeds et al., 2012). Nineteen stilbenes have been isolated in *Cannabis* with characteristic structural backbones such as spirans, phenanthrenes and bibenzyls (Flores-Sanchez and Verpoorte, 2008). They include molecules such as cannabistilbene I, IIa and IIb, as well as dihydroresveratrol. Interestingly, bibenzyl stilbenes, including the putative 3-*O*-methylbatatasin, were strongly induced in *Cannabis* leaves by UV radiations (Marti et al., 2014).

### Biosynthetic Pathway Leading to the Different Classes of Phenolic Compounds

Phenolic compounds are produced through the phenylpropanoid pathway in the cytoplasm and are subsequently transported in the vacuole or deposited in the cell wall (**Figure 2**). Routes to the major classes of phenolic compounds involve (i) the core phenylpropanoid pathway from phenylalanine to an activated (hydroxy) cinnamic acid derivative (p-coumaroyl CoA), via the actions of the phenylalanine-ammonia-lyase (PAL), cinnamate 4-hydroxylase (C4H, a cytochrome P450) and 4-coumarate-CoA ligase (4CL), as well as specific branch pathways for the formation of (ii) simple esters, lignins and lignans, (iii) flavonoids, (iv) coumarins, and (v) stilbenes (Andre et al., 2009; Naoumika et al., 2010; Docimo et al., 2013) (**Figure 2**). Although the flavonoid pathway has been extensively studied in several plants, there is no specific data on the biosynthesis of flavonoids in *Cannabis*. Generally, lignans such as secoisolariciresinol are produced *in planta* by stereoselective coupling of coniferyl alcohol moieties, via two distinct dirigent proteins, giving rise to (+) or (-) pinoresinol. Each pinoresinol can then be further enantiospecifically reduced to lariciresinol and secoisolariciresinol (Dalisay et al., 2015). The key molecular events associated with the biosynthesis of lignanamides are still unknown. The structure of these molecules suggests, however, a condensation of the precursors tyramine and CoA-esters of coumaric, caffeic, and coniferic acid (Flores-Sanchez and Verpoorte, 2008), followed by an oxidative coupling reaction catalyzed by a dirigent protein, as described for lignans. The flavonoid pathway is initiated by condensation of p-coumaroyl CoA with three molecules of malonyl-CoA (**Figure 2**). Naringenin chalcone is rapidly isomerized by the enzyme chalcone isomerase (CHI) to form naringenin, the branch point of flavonols on one hand and flavones on the other one. Flavanone 3-hydroxylase (F3H) may subsequently hydroxylate naringenin to produce the dihydroflavonol, dihydrokaempferol, which can be further hydroxylated by flavonoid 3′ hydroxylase (F3′H) to form dihydroquercetin. Dihydrokaempferol and dihydroquercetin are substrates of flavonol synthase (FLS), which catalyzes the production of the flavonols kaempferol and quercetin, respectively. Naringenin may alternatively be converted to apigenin, by a reaction catalised by a flavone synthase (FNS). Apigenin can be further hydroxylated by a flavonoid 3′ hydroxylase (F3′H) to form luteolin which is likely the precursor of the diverse cannflavins (Flores-Sanchez and Verpoorte, 2008).

### Health Benefits Associated with Phenolic Compounds

In plants, phenolic compounds may act as antioxidants under certain physiological conditions and, thereby, protect plants against oxidative stress. In humans, it was shown that there is a correlation between dietary phenolic compound intake and a reduced incidence of chronic diseases such as cancers, cardiovascular and neurodegenerative diseases (Arts and Hollman, 2005), but these positive health effects may not be entirely explained by the phenolic antioxidant properties, as they are poorly bioavailable. Phenolic compounds may induce the up-regulation of endogenous antioxidant enzymes *in vivo*, due to their ability to act as pro-oxidants and generate Reactive Oxygen Species (ROS) (Halliwell et al., 2005). They may also exert their action through non-specific protein binding interactions (Gertsch et al., 2010). The flavones and flavonols found in *Cannabis* exert a wide range of biological effects, including properties shared by terpenes and cannabinoids. They present anti-inflammatory, anti-cancer and neuro-protective properties as reviewed in (Andre et al., 2010). In addition, apigenin has been shown to possess anxiolytic (Murti et al., 2012) and oestrogenic properties (Wang and Kurzer, 1998). The specific cannflavin A et B are potent anti-inflammatory compounds, via inhibition of prostaglandin E2 and 5-lipoxygenase (Werz et al., 2014). Health-related studies concerning lignanamides are scarce and showed *in vitro* anti-inflammatory (Sun et al., 2014) and cytotoxic activities (Cui-Ying et al., 2002). Lignans in general show a wide array of health-promoting properties including antioxidant, antiviral, antidiabetic, antitumorigenic and anti-obesity activities. Interestingly, secoisolariciresinol, lariciresinol and pinoresinol are converted into enterolignans by the anaerobic intestinal microflora, which are mammalian oestrogen precursors (phyto-oestrogens) (Wang et al., 2010). Due to the structural similarity of enterolignans with mammalian oestrogens, these compounds are potentially interesting for combating some hormone-dependent cancers. The mechanisms of action of the lignans are, however, complex, with multiple targets involved (Sainvitu et al., 2012).

## Synergistic and Antagonistic Effects Between Phytochemicals

It is now well accepted that the health benefits of fruits, vegetables and other plant foods are due to the synergy or interactions between the different bioactive compounds or other nutrients present in the whole foods, and not to the action of a sole compound (Liu, 2013). Similarly, *Cannabis*-based therapeutics exert their pharmacological effects in humans via synergistic or antagonistic interactions between the various phytochemicals described above. These interactions may occur through various mechanisms including: (i) bioavailability, (ii) interference with cellular transport processes, (iii) activation of pro-drugs or deactivation of active compounds to inactive metabolites, (iv) action of synergistic partners at different points of the same signaling cascade (multi-target effects) or (v) inhibition of binding to target proteins (Efferth and Koch, 2011). A good example is the stronger muscle-antispastic effect of a *Cannabis* extract compared to pure THC, which represents an important finding for the treatment of multiple sclerosis (Wagner and Ulrich-Merzenich, 2009). Non-THC cannabinoids have shown positive influence on the side effects induced by THC such as anti-anxiety activities. CBD may also reduce the induced cognitive and memory deficits in subjects smoking *Cannabis* (Wright et al., 2013). CBD affects the pharmacokinetics of THC through different mechanisms: (i) by fluidizing the membranes and therefore increasing the penetration of THC in muscle cells, and (ii) by inhibiting the P450-mediated hepatic drug metabolism, which is involved in the degradation and elimination of the molecule (Klein et al., 2011). Terpenes may also alter the pharmacokinetics of THC by increasing the blood-brain barrier permeability. This characteristic has notably been used to patent a transdermal patch, which delivers cannabinoids into the bloodstream by using a terpene as a permeation agent (Smith, 2015). Terpenes may also modulate the affinity of THC for the CB1 receptor and interact with neurotransmitter receptors, which may support contributions of terpenes on cannabinoid-mediated analgesic and psychotic effects (McPartland and Russo, 2001; Russo, 2011). In view of the potential of phytocannabinoid-terpene synergy, it has been suggested to tailor novel therapeutic treatments such as CBD-terpene extracts to be used against acne, MRSA, depression, anxiety, insomnia, dementia and addiction (Russo, 2011).

Flavonoids may also modulate the pharmacokinetic of THC, via inhibition of the hepatic P450 enzymes (3A11 and 3A4) (McPartland and Russo, 2001; Russo, 2011).

Finally, there is an example of predator-targeted synergy between terpenes and phytocannabinoids in the *Cannabis* plant itself: on one side, the specific mixture of monoterpenes and sesquiterpenes determines viscosity and thereby the stickiness of *Cannabis* exudations necessary to trap the insects, and on the other one, the phytocannabinoid acid acts as potent insecticidal molecules (Sirikantaramas et al., 2005; Russo, 2011).

## *Cannabis* Trichomes: Small Factories of Phytochemicals

Trichomes are epidermal protuberances covering the leaves, bracts and stems of plants and some of them, like the glandular trichomes, are capable of secreting (or storing) secondary metabolites as a defense mechanism. Several papers have focused on the characterization of these specialized structures using *-omics* (Wang et al., 2009a; Schilmiller et al., 2010; McDowell et al., 2011; Jin et al., 2014), because their integrated study can favor the development of technologies harnessing their rich biochemical potential (Schilmiller et al., 2008). An *-omics* database (TrichOME; available at: http://www.planttrichome.org/) enabling comparative analyses in plant trichomes has also been created with the purpose of providing the researchers with the possibility to mine data relative to metabolites, genes, expression profiles (Dai et al., 2010). Additionally, several procedures (in some instances supported by a video demonstration; e.g., Nayidu et al., 2014) for the isolation of trichomes from the leaves of different plant species are available (e.g., Marks et al., 2008; Balcke et al., 2014).

**FIGURE 3:**
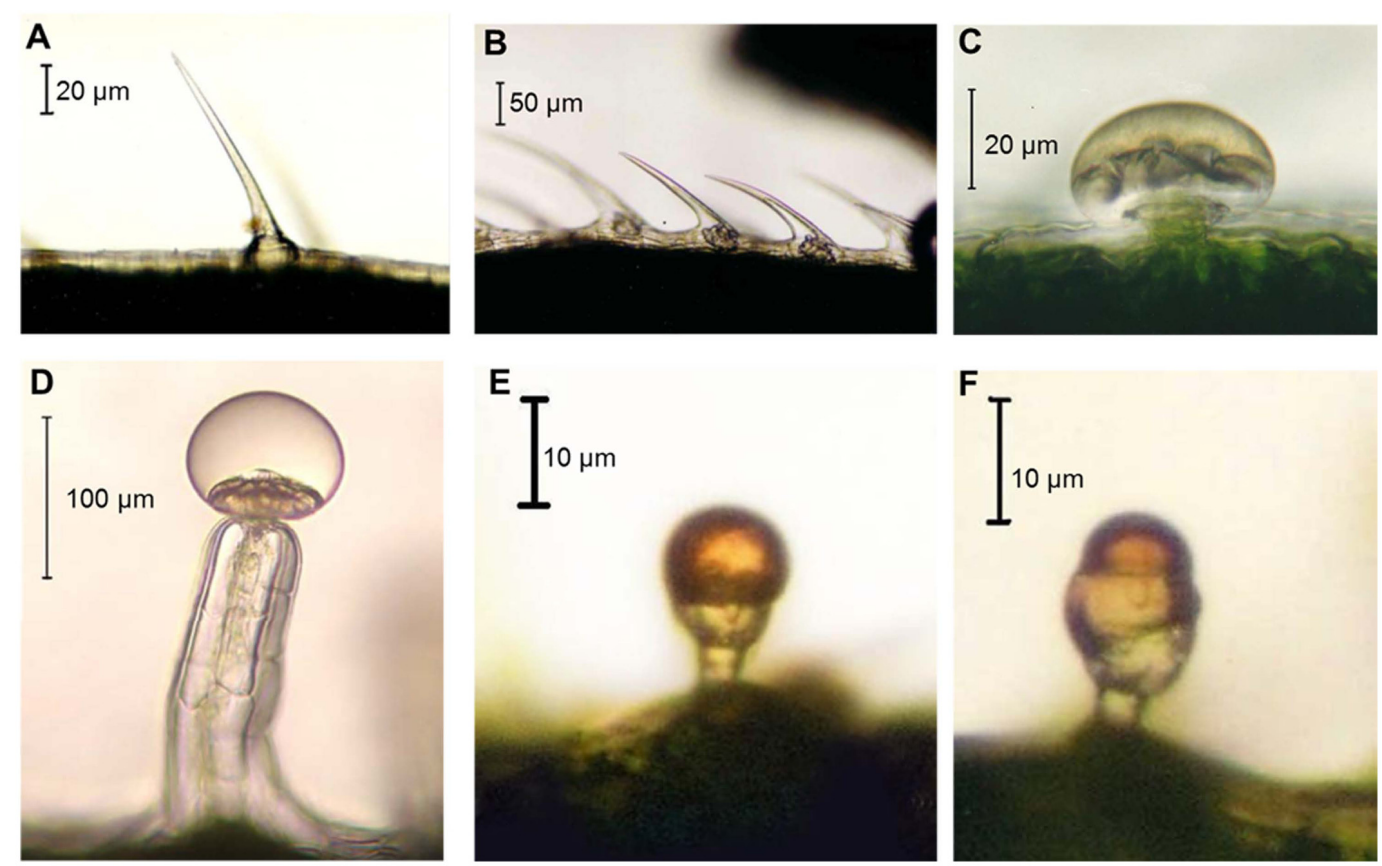
**Hemp trichome types. (A)** Unicellular non-glandular trichome; **(B)** cystolythic trichomes; **(C)** capitate sessile trichome; **(D)** capitate-stalked trichome; **(E)** simple bulbous trichome; **(F)** complex bulbous trichome. Images kindly provided by Dr. David J. Potter.

Hemp has different types of trichomes (**Figures 3A–F**) which belong to two categories, i.e., glandular and non-glandular (Happyana et al., 2013). Capitate sessile, capitate stalked and bulbous hemp trichomes are secretory structures (**Figures 3C–F**).

In *Cannabis* THCA is accumulated in the heads (glands) of both capitate-stalked and capitate sessile trichomes, but in the former the content is higher (Mahlberg and Kim, 2004). Notably, in the textile variety, the cannabinoids CBDA and CBCA occur at high concentrations instead of THCA, while the reverse is true for drug strains (Mahlberg and Kim, 2004).

Studies on hemp have demonstrated that THCA is synthesized in the storage cavity and that the enzyme responsible for THCA production, i.e., THCAS, follows a sorting pathway from the secretory cells to the storage cavity (Sirikantaramas et al., 2005). The accumulation in the storage cavity is due to the cytotoxicity of cannabinoids: they induce indeed death via apoptosis, when supplied for 24 h to both hemp and tobacco cell suspension cultures (Sirikantaramas et al., 2005). Heterologous expression of THCAS fused to GFP in tobacco leads to fluorescence of the trichome heads, thereby confirming the localization of the enzyme in the storage cavity (Sirikantaramas et al., 2005).

Depending on their color, hemp glandular trichomes show different secretory phases (Mahlberg and Kim, 2004): the mature secreting gland appears translucent (at this stage the cannabinoid content is the highest), while aging glands are yellow and senescing brown.

According to the current model cannabinoids are produced via terpenes secreted by plastids present in the disk cells and phenols stored in their vacuole (Mahlberg and Kim, 2004): analyses using the electron microscope have shown that oily secretions (most likely terpenes) round in shape are secreted from the plastids (which have the appearance of reticulate bodies). Subsequently vesicles are released into the cavity together with fibrillar matrix originating from the cell walls of the disk cells. The fibrillar matrix is transported to the subcuticular cell wall and contributes to its thickening via yet unidentified mechanisms (Mahlberg and Kim, 2004).

Besides cannabinoids, *Cannabis* trichomes produce other secondary metabolites, namely terpenes (see previous paragraph on *Cannabis* phytochemicals), which are responsible for the typical plant aroma (Russo, 2011). Among the *Cannabis* terpenes of low abundance, is nerolidol (0.09% of the total terpene content, Ross and ElSohly, 1996), which, interestingly, has anti-malarial and anti-leishmanial effects (reviewed by Russo, 2011). Given the pharmacological importance of these compounds, it would be interesting to devise engineering strategies aiming at either boosting the secondary metabolism, or increasing the density of trichomes in *Cannabis*. Among the possible genetic engineering approaches, it is here worth mentioning two examples recently reported in *Artemisia annua*. We will here discuss only these two examples, as further discussion on how to scale up the production of cannabinoids is presented later in this review.

It has been recently shown that the transformation of *A. annua* with the *rolB* and *rolC* genes of *Agrobacterium rhizogenes* led to plants with an increased content of artemisinin (Dilshad et al., 2015). The *rol* genes are known for their stimulatory action on plant secondary metabolism (Bulgakov, 2008). The study on *A. annua* showed that *rolB* and *rolC* trigger different effects, with *rolB* showing enhanced production with respect to *rolC*. An additional study on *A. annua* has shown that the expression of a β-glucosidase from *Trichoderma reesei* increases glandular trichome density and artemisinin production (Singh et al., 2015). The hydrolytic enzyme favors the release of active plant growth regulators from the conjugates stored in the plastids, thereby favoring trichome formation, as well as biomass production and leaf area (Singh et al., 2015). It would be interesting to devise an engineering strategy aimed at increasing the density of trichomes in *Cannabis*, by adopting a similar strategy. *–Omics* studies on *Cannabis* trichomes will help identify important genes, among which transcription factors (involved in trichome formation), which can be likewise used for engineering approaches.

## *Cannabis* Biotechnology: Challenges and Prospects

*Cannabis* is a precious plant with multiple applications, hence the possibility of engineering it genetically to produce useful compounds/raw products is highly valuable. In this section of the review we will: (i) discuss the progress made in *Cannabis in vitro* propagation together with the biotechnological prospects of *Cannabis* genetic engineering, by highlighting the challenges and benefits, (ii) describe the hairy root culture system as a tool for the scalable production of cannabinoids and (iii) discuss the advantages of the *Cannabis* cell suspension culture system.

### *Cannabis In Vitro* Propagation and Transformation

The cultivation of *Cannabis* is severely regulated in many countries; therefore alternative *in vitro* growth techniques are receiving a lot of attention. The *in vitro* cultivation of *Cannabis* is also an advantageous way to preserve cultivars/clones (Lata et al., 2009a) with specific metabolite signatures.

Methods to multiply *C. sativa* plants *in vitro* via stimulation of axillary buds on nodal segments, or induction of adventitious buds in the shoot tips have been described (Lata et al., 2009a; Wang et al., 2009b). It was shown that micro-propagated plants are genetically stable; therefore the method is appropriate and useful for the clonal multiplication of this important crop (Lata et al., 2010).

A protocol has also been developed for the propagation of hemp via the synthetic seed technology. According to this procedure, axillary buds or nodal segments are encapsulated in calcium alginate beads (Lata et al., 2009b, 2011), which can then be stored and subsequently used for clonal propagation of the plant. This system was shown to allow the successful growth of homogeneous and genetically stable *Cannabis* plants even after 6 months of storage (Lata et al., 2011).

To set up a successful *Cannabis* transformation protocol, the mastery of *in vitro* culture techniques is necessary: whether the strategy adopts plant explants or undifferentiated calli as starting material, the regeneration of the whole plant is a mandatory step. Organ regeneration, in particular shoots, can be quite cumbersome and therefore the screening of different plant growth regulator concentrations and combinations has to be carried out to find the right culture medium composition.

*Cannabis sativa* is a notorious recalcitrant plant to transformation, because the regeneration efficiencies are quite low and dependent upon the cultivar, tissue, plant age and growth regulator combination (Slusarkiewicz-Jarzina et al., 2005). As an example, although successful transformation of hemp calli via *Agrobacterium tumefaciens* was reported by Feeney and Punja (2003), the undifferentiated cells failed to regenerate the shoots. The cells were transformed with phosphomannose isomerase and colorimetric assays showed successful expression of the transgene.

Nevertheless some success in hemp regeneration was reported and shown to be linked to the choice of specific plant growth regulators. For example the addition of thidiazuron (TDZ), which has cytokinin-like activity, was shown to increase the development of shoots in hemp explants (Lata et al., 2009a) and in leaf-derived calli of a high yielding THCA clone (Lata et al., 2010). The herbicide DICAMBA was also reported to favor the regeneration of hemp shoots from calli (Slusarkiewicz-Jarzina et al., 2005).

*Cannabis* transformation protocols using plant explants (thereby avoiding the passage to undifferentiated cells) have been described for several important crops (e.g., cotton, Zapata et al., 1999; jute, Saha et al., 2014). Notably, successful transformation of hemp plants was reported by MacKinnon et al. (2001) using shoot tips: the protocol uses shoot tip explants and the regeneration potential of the shoot apical meristem after infection with *A. tumefaciens*. Additionally a patent application was filed describing *Cannabis* transformation using 1–2 cm hypocotyl explants, the plant growth regulators zeatin and 6-benzylaminopurine (BAP) for shoot regeneration (Sirkowski, 2012).

### Hairy Root Cultures for the Production of Cannabinoids

An additional system offering interesting applications for the industrial production of compounds showing pharmaceutical effects in humans is the hairy root system, a type of *Agrobacterium*-transformed plant tissue culture used to study plant metabolic processes. Transformation of hemp and subsequent establishment of hairy root culture has been described by Wahby et al. (2013) using both *A. rhizogenes* and *A. tumefaciens*. In this study hypocotyls were found to be the most responsive tissue for infection. The hairy root system is very interesting for the production of secondary metabolites in medicinal plants (Jiao et al., 2014; Patra and Srivastava, 2014; Wawrosch et al., 2014; Gai et al., 2015; Tian, 2015) or to engineer model plants to secrete industrially valuable metabolites. For example, in tobacco transgenic hairy roots the production of THCA was successfully obtained by expressing hemp THCAS (Sirikantaramas et al., 2007). The hairy root system is characterized by hormone-independent high growth rate and by the same metabolic potential as the original organ (Pistelli et al., 2010). A protocol for the establishment of hairy roots from *Cannabis* callus cultures has also been described (Farag and Kayser, 2015). In this study calli were grown on full-strength B5 medium supplemented with 4 mg/L 1-Naphthaleneacetic acid (NAA) and their potential of cannabinoid production was evaluated. The authors found that after 28 days of cultivation in the dark, a peak could be observed in the accumulation of cannabinoids in culture media supplemented with different concentrations of indole-3-acetic acid (IAA). However, the yield remained below 2 μg/g of dry weight, thereby showing that further optimizations are still required in this field. The induction of rhizogenesis in undifferentiated *Cannabis* cells is important, because it can be performed on calli overexpressing key transcription factors and/or genes involved in the cannabinoid pathway.

The production of cannabinoids in hemp hairy root cultures can be then further implemented with adsorbents to avoid toxicity issues (a more detailed discussion concerning possible ways to avoid toxicity is present in the section dedicated to heterologous plant hosts). In alternative, inducible promoters can be used, like for instance the glucocorticoid-inducible promoter, which was already shown to be effective in inducing a controlled, reversible and dosage-dependent expression of GFP in *Catharanthus roseus* hairy roots (Hughes et al., 2002).

### *Cannabis* Cell Suspension Cultures for the Production of Cannabinoids

Plant cell suspension cultures offer important advantages, as they can be transformed and then cultivated in bioreactors for the production of useful metabolites (Weathers et al., 2010; Bortesi et al., 2012; Liu et al., 2012; Han et al., 2014). *Cannabis* callus cultures are not able to produce any cannabinoids, irrespective of the chemotypes (drug-, hybrid-, or fiber-type) used as mother plants or growth regulators used in the culture medium (Pacifico et al., 2008). The transformation of hemp cell suspension cultures with genes involved in specific metabolic pathways can offer the possibility of enhancing the production of important classes of metabolites such as cannabinoids but also of others with potential pharmacological use. In this paragraph we will discuss about potential biotechnological approaches to boost the production of cannabinoids in *Cannabis* cell suspension culture.

**FIGURE 4:**
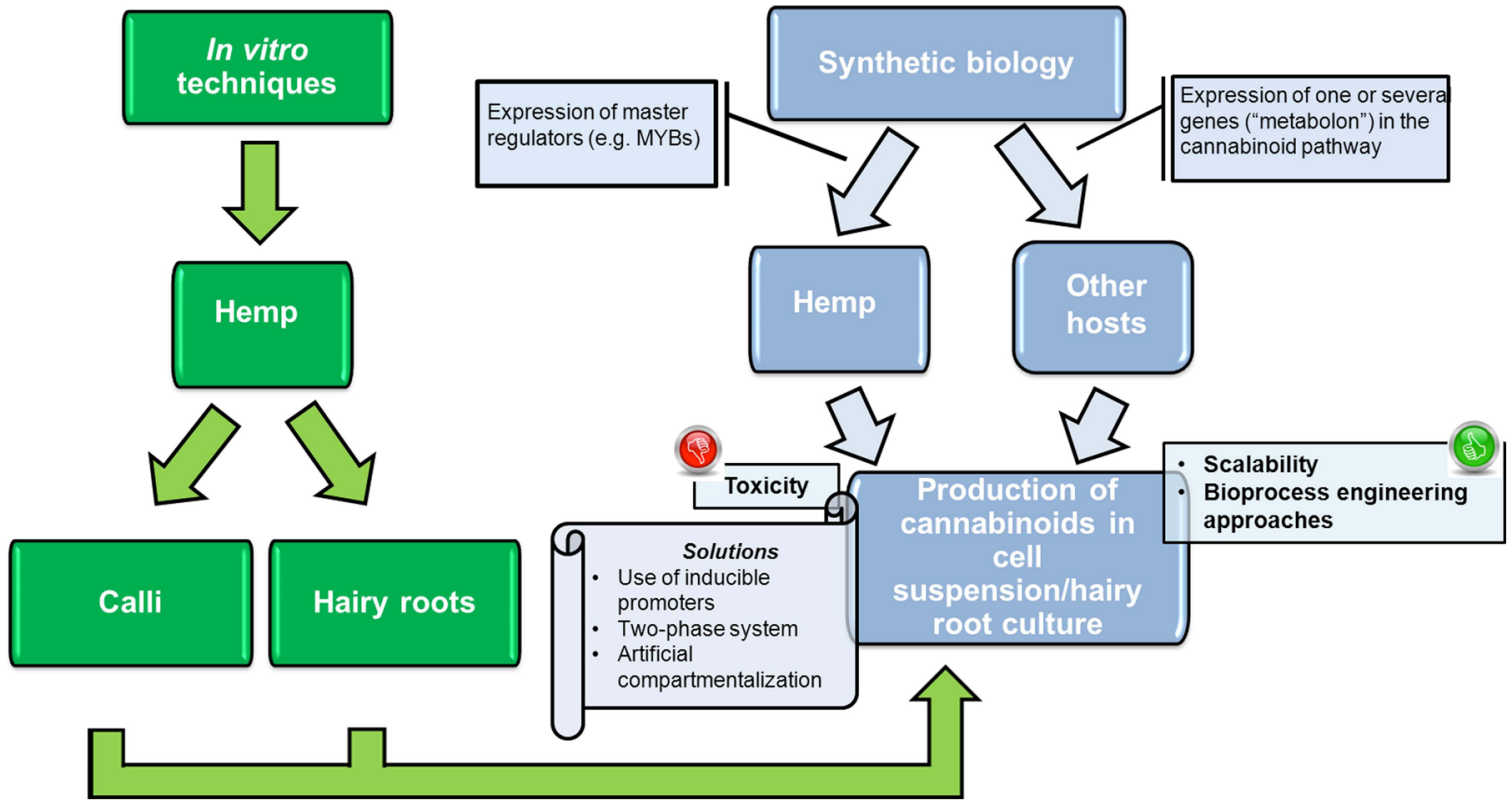
**Workflow showing the achievements (in green) and potential future approaches (in light blue) to produce cannabinoids in cultures of *Cannabis*, as well as other plant hosts**.

The increased production of cannabinoids in *Cannabis* cell suspension cultures can be achieved via the expression of transcription factors involved in *Cannabis* gland biochemistry (**Figure 4**). Transcription factors represent a powerful tool in plant metabolic engineering, because of their “cascade” mechanism of action: if master regulators involved in cannabinoid biosynthesis are identified in *C. sativa* trichomes, they could be expressed constitutively or inducibly in *Cannabis* cell suspension cultures. It is important to mention here that two transcription factors belonging to the MYB family were already shown to be preferentially expressed in *Cannabis* glands (Marks et al., 2009) and therefore represent ideal candidates to express. These genes show homology with *Arabidopsis thaliana* MYB112 and MYB12, which are known to be involved in the tolerance to oxidative stress and flavonol biosynthesis, respectively (Marks et al., 2009 and references therein). The expression of these transcription factors in an inducible manner is a strategy worth being tested for the production of cannabinoids. The inducible expression will limit the negative effects caused by the toxicity of the accumulating cannabinoids during the growth of the transformed plant cells (as more thoroughly described in the next section).

In addition to the genetic engineering approach, plant cell suspension cultures can be elicited to boost the production of secondary metabolites. The literature is rich in examples concerning the increased expression of secondary metabolites in plant cells elicited with different factors (reviewed recently by Ncube and Van Staden, 2015). Both biotic and abiotic stress factors can indeed be used to re-direct the plant metabolism: nutrients, light, temperature, fungal elicitors are among the most common factors manipulated.

In hemp suspension cells, elicitation with biotic and abiotic elicitors did not induce an increase in cannabinoids (Flores-Sanchez et al., 2009); however, jasmonic acid was shown to elicit the production of the antioxidant tyrosol (Pec et al., 2010).

It is here worth mentioning the effect of a so far neglected element, silicon (Si). Despite being a non-essential element for plant growth, Si is known to increase plant vigor and to alleviate the effects of exogenous stresses (Epstein, 2009). Very recently Si was shown to alleviate the effects of salt stress and to induce the production of chlorogenic acid in *Lonicera japonica* (Gengmao et al., 2015). Given the stimulatory effects that Si has on plant metabolism, it is interesting to further investigate, from a molecular perspective, the effects of Si supplementation on *Cannabis* secondary metabolite production. Cyclodextrins have also been used in plant cell suspension cultures to enhance the production of various non-polar metabolites such as stilbenes (Yang et al., 2015), phytosterols (Sabater-Jara and Pedreño, 2013) or triterpenes (Goossens et al., 2015). Cyclodextrins are cyclic oligosaccharides consisting of five or more α-D-glucopyranose residues. They are known to form inclusion complexes with lipophilic compounds, including cannabinoids (Hazekamp and Verpoorte, 2006), in their hydrophobic cavity, thereby improving metabolite solubility in an aqueous environment. In addition, cyclodextrins, thanks to their chemical structure similar to that of the alkyl-derived oligosaccharides released from plant cell wall when a fungal infection occurs, act as elicitors of secondary metabolite production (Sabater-Jara and Pedreño, 2013).

It would therefore be worth investigating the effect of cyclodextrins on the production of the non-polar cannabinoids in hemp suspension cell cultures.

## Cannabinoid Production in Heterologous Plant Hosts: How it can be Achieved and what Should be Taken into Account

The expression of genes involved in the cannabinoid biosynthetic pathway in cell suspension cultures of plants other than *Cannabis* represents an interesting alternative for the scalable production of cannabinoids (**Figure 4**). For example synthetic biology could be used to recreate the cannabinoid biosynthetic pathway in heterologous plant cells via the expression of THCAS, together with the upstream enzymes involved in the synthesis of CBG, i.e., the tetraketide synthase (the type III PKS), the aromatic prenyltransferase and the OAC (Gagne et al., 2012). In this respect tobacco bright yellow 2 (BY-2) cells are very interesting expression hosts, given their wide use in plant biotechnology as “workhorse” for the production of recombinant proteins (e.g., Reuter et al., 2014).

The biomimetic production of cannabinoids in heterologous plant hosts is challenging, however, one strategy that is worth taking into account concerns the use of synthetic “metabolons” (Singleton et al., 2014). A “metabolon” is the association of enzymes which carry out a series of sequential reactions in a given pathway. Examples for the occurrence of metabolons exist in plants for pathways involving, e.g., the synthesis of phenylpropanoids (Chen et al., 2014) and the cyanogenic glycoside dhurrin (Nielsen et al., 2008). Entire metabolic pathways can be engineered via the use of synthetic metabolons enabling the association of enzymes in close proximity: this allows a more efficient shunting of intermediates at the active site of enzymes acting in chain (Singleton et al., 2014). One possible way to assemble a synthetic metabolon is via the use of a scaffolding protein enabling the association of the enzymes (Singleton et al., 2014; Pröschel et al., 2015). In the specific case of cannabinoid production, the creation of a synthetic metabolon comprising for instance the type III PKS and OAC (Gagne et al., 2012), together with the aromatic prenyltransferase and the THCAS, can be achieved via (i) the use of dockerin-cohesin modules, or (ii) the metazoan signaling proteins SH3-, PDZ-, GBD binding domains, or (iii) the SpyTag/SpyCatcher domains (recently reviewed by Pröschel et al., 2015).

The assembly of multimodular constructs for expression in plants is no longer an insurmountable challenge, thanks to the development of methods like the Gateway-mediated cloning (reviewed by Dafny-Yelin and Tzfira, 2007), Golden Gate (Binder et al., 2014), GoldenBraid (Sarrion-Perdigones et al., 2011), to name a few.

When cannabinoids are produced in heterologous plant hosts, toxicity effects have to be taken into account, as it was shown that THCA and CBGA cause cell death via apoptosis in cells of *Cannabis* and tobacco BY-2 (Sirikantaramas et al., 2005). For plant cell suspension cultures cultivated in bioreactors, the *in situ* product removal via a two-phase culture system might be useful to favor the accumulation of the toxic metabolites produced in sites which are separated from the cells (Cai et al., 2012) (**Figure 4**). The use of adsorbents in the culture medium can not only sequester the toxic compounds, but also stimulate the secondary metabolite biosynthesis (Cai et al., 2012 and references therein).

One additional approach that can be used to avoid product toxicity in plant cell suspension cultures is artificial compartmentalization (**Figure 4**). This approach has been recently proposed in *A. annua* cell cultures for the production of artemisinin (Di Sansebastiano et al., 2015). The authors induced the formation of an artificial compartment (generated by membranes deriving from endocytosis and the endoplasmic reticulum-vacuole trafficking) via the expression of a truncated SNARE protein, AtSYP51. The creation of an artificial compartment can be used for the production of cannabinoids, because it can trap and stabilize the toxic secondary metabolites until extraction is performed, in a manner analogous to what discussed for artemisinin.

## Perspectives and Conclusion

Hemp is a unique versatile plant, which can provide high biomass quantities in a short time. Hemp stem is used as a source of woody and bast fibers for the construction and automotive industries, while hemp seeds are used as a source of dietary oil and hemp leaves and flowers as a source of bioactive components.

To date, more than 540 phytochemicals have been described in hemp (Gould, 2015), and their pharmacological properties appear to go much beyond psychotic effects, with the capacity to address needs like the relief of chemotherapy-derived nausea and anorexia, and symptomatic mitigation of multiple sclerosis.

Continuously discovering new prototypes of drugs is of tremendous importance to meet tomorrow’s challenges in terms of public health (Atanasov et al., 2015). Nature has already provided a large source of new molecules and new skeletons. A recent review reporting the new drugs available on the market during the last 30 years showed that more than 35% of these new drugs have a direct natural origin. This percentage rises to over 60% if we take into account all the drugs whose structure is inspired by a natural pharmacophore (Newman and Cragg, 2012). *Cannabis* presents a colossal potential for enlarging the library of bioactive metabolites. Compounds can be obtained from hemp trichomes, cell suspension cultures, hairy root systems, or via the biotransformation of THCA or CBDA using fungal, bacterial, or plant cells (Akhtar et al., 2015).

Our increasing knowledge on the key molecular components triggering the diverse phytochemical pathways *in planta* (**Figure 2**), may also allow, through a genetic engineering approach, to further increase the production of specific cannabinoids, terpenes, or phenolic compounds, or to reconstruct the pathway in heterologous systems using a synthetic biology approach. Apart from the importance of studies focused on improving *Cannabis* genetic transformation, it is necessary to know more about the regulatory mechanisms involved in secondary metabolite production in *C. sativa*. For example enzymological and structural studies will help devise protein engineering approaches to improve the catalytic functions of key enzymes (Taura et al., 2007a). However, further studies would still be needed to elucidate other key genes involved in biosynthetic pathways of, for instance, less-abundant cannabinoid derivatives. For that purpose, the combination of metabolomics with genome-based functional characterizations of gene products would provide an accelerated path to discovering novel biosynthetic pathways to specialized metabolites. Indeed, the functions of numerous genes have been identified and characterized through the correlation of gene expression and metabolite accumulation (Sumner et al., 2015). Classical approaches used focused on the spatial and temporal distribution of the targeted phytochemicals and on the plant transcriptome, as influenced by the developmental stage and environmental stresses. With respect to the resurgence of interest in *Cannabis* phytochemicals nowadays, the results of such studies will be soon available.

## Author Contributions

CA was involved in the review writing, J-FH was involved in manuscript refinement, and GG initiated the idea of the review and was involved in the manuscript writing.

## Conflict of Interest Statement

The authors declare that the research was conducted in the absence of any commercial or financial relationships that could be construed as a potential conflict of interest.
